# The Impact of Molecular Subtyping on Pathological Staging of Pancreatic Cancer

**DOI:** 10.1097/SLA.0000000000005050

**Published:** 2023-01-10

**Authors:** Stephan B. Dreyer, Sarah Rae, Kirsty Bisset, Rosie Upstill-Goddard, Georgios Gemenetzis, Amber L. Johns, Australian Pancreatic Cancer Genome Initiative, Glasgow Precision Oncology Laboratory, Euan J. Dickson, Anubhav Mittal, Anthony J. Gill, Fraser Duthie, Antonio Pea, Rita T. Lawlor, Aldo Scarpa, Roberto Salvia, Alessandra Pulvirenti, Alessandro Zerbi, Federica Marchesi, Colin J. McKay, Andrew V. Biankin, Jaswinder S. Samra, David K. Chang, Nigel B. Jamieson

**Affiliations:** * Wolfson Wohl Cancer Research Centre, Institute of Cancer Sciences, University of Glasgow, Garscube Estate, Switchback Road, Bearsden, Glasgow, Scotland, United Kingdom;; † West of Scotland Pancreatic Unit, Glasgow Royal Infirmary, Glasgow, United Kingdom;; ‡ The Kinghorn Cancer Centre, 370 Victoria Street, Darlinghurst and Garvan Institute of Medical Research, Sydney, NSW, Australia;; § Department of Surgery, Royal North Shore Hospital, St Leonards, Sydney, NSW, Australia;; || University of Sydney, Sydney, NSW, Australia;; ¶ Cancer Diagnosis and Pathology Group Kolling Institute of Medical Research and Department of Anatomical Pathology, Royal North Shore Hospital, Sydney, NSW, Australia;; # Northern Clinical School, Faculty of Medicine, University of Sydney, Sydney, Australia;; ** Department of Pathology, Queen Elizabeth University Hospital, Glasgow, United Kingdom;; †† General and Pancreatic Surgery Department, Pancreas Institute, University and Hospital Trust of Verona, Verona, Italy;; ‡‡ ARC-Net Research Center, University and Hospital Trust of Verona, Verona, Italy;; §§ Department of Diagnostics and Public Health, University and Hospital Trust of Verona, Verona, Italy; Pancreatic Surgery Unit, IRCCS Humanitas Research Hospital, Rozzano, Milan, Italy;; ¶¶ Department of Biomedical Sciences, Humanitas University, Pieve Emanule, Milan, Italy;; ## Department of Immunology, IRCCS Humanitas Research Hospital, Rozzano, Italy; and; *** Department of Medical Biotechnology and Translational Medicine, University of Milan, Milan, Italy.

**Keywords:** cancer staging, molecular subtyping, pancreatic cancer

## Abstract

The long-term outcomes following surgical resection for pancreatic ductal adenocarcinoma (PDAC) remains poor, with only 20% of patients surviving 5 years after pancreatectomy. Patient selection for surgery remains suboptimal largely due to the absence of consideration of aggressive tumor biology.

**Objective:**

The aim of this study was to evaluate traditional staging criteria for PDAC in the setting of molecular subtypes.

**Methods:**

Clinicopathological data were obtained for 5 independent cohorts of consecutive unselected patients, totaling *n* = 1298, including *n* = 442 that underwent molecular subtyping. The main outcome measure was disease-specific survival following surgical resection for PDAC stratified according to the American Joint Commission for Cancer (TNM) staging criteria, margin status, and molecular subtype.

**Results:**

TNM staging criteria and margin status confers prognostic value only in tumors with classical pancreatic subtype. Patients with tumors that are of squamous subtype, have a poor outcome irrespective of favorable traditional pathological staging [hazard ratio (HR) 1.54, 95% confidence interval (CI) 1.04–2.28, *P* = 0.032]. Margin status has no impact on survival in the squamous subtype (16.0 vs 12.1 months, *P* = 0.374). There were no differences in molecular subtype or gene expression of tumors with positive resection margin status.

**Conclusions:**

Aggressive tumor biology as measured by molecular subtype predicts poor outcome following pancreatectomy for PDAC and should be utilized to inform patient selection for surgery.

Pancreatic ductal adenocarcinoma (PDAC) has overtaken breast cancer as the 3^rd^ most common cause of cancer related death in Western societies, and is predicted to be 2^nd^ by 2025.^1,2^

Transcriptomic molecular subtyping of PDAC has consistently identified a subgroup, termed squamous (also known as basal) subtype, characterized by epigenetic changes that drive immune evasion and epithelial-to-mesenchymal transition as compared to the classical pancreatic subtype.^3–5^ The distinct molecular features underpinning the squamous subtype is associated with aggressive tumor biology and a poorer outcome. Although these features may potentially provide novel targets for subtype-specific vulnerabilities and ultimately direct therapy for PDAC, they are not yet utilized clinically to inform prognosis.^3,6^

Pathological staging of resected PDAC has been enhanced through modifying the American Joint Commission for Cancer (AJCC) staging system (8^th^ Edition),^7^ and resection margin involvement is established as a powerful predictor of poor outcome.^8–11^ Although the prognostic value of traditional pathological staging criteria including margin status^9^ has been validated in several studies,^7,12^ these traditional histopathological features have not been rigorously assessed according to molecular subtype.

Incorporating molecular characterization of PDAC is vital for individualized outcome prediction, therapy allocation, clinical trial eligibility, and to facilitate result comparison across studies and institutions. In this study, using 5 independent highly annotated multi-institutional cohorts, we sought to validate the AJCC 8^th^ edition staging criteria, and determine the value of resection margin involvement in the context of established PDAC molecular subtypes, in patients following resection for pancreatic cancer.

## Methods

### Patient Cohort Description

Clinicopathological and complete outcome data were obtained from prospectively maintained independent cohorts of patients with resected PDAC. Patients were accrued prospectively for the Australian Pancreatic Cancer Genome Initiative (APGI) cohort (www.pancreaticcancer.net.au) as part of the International Cancer Genome Consortium (ICGC; www.icgc.org).^13^ Additional cohorts were recruited from the West of Scotland Pancreatic Unit, Glasgow Royal Infirmary, United Kingdom; the Royal North Shore Hospital (RNSH), Sydney, Australia; ARC-Net biobank and The Pancreas Institute, University and Hospital Trust of Verona, Italy; and Pancreatic Surgery Unit of Humanitas Hospital in Milan, Italy (Supplementary table 1, http://links.lww.com/SLA/D251). Patients with oligometastatic disease were excluded. Ethical approval for the acquisition of data and biological material was obtained from the Human Research Ethics Committee at each participating institution (supplementary data, http://links.lww.com/SLA/D251).

**Table 1:** Molecular Subtyped Cohort Patient Characteristics

	All Patients			Classical Pancreatic			Squamous		
	*n* = 442	Median DSS	*P*	*n* = 330	Median DSS	*P*	*n* = 112	Median DSS	*P*
Variables	No. (%)	mo	(*Log-Rank*)	No. (%)	mo	*(Log-RANK)*	no. (%)	mo	*(Log-Rank)*
Sex									
Male	230 (52.0)	19.6	0.158	171 (38.7)	22.0	0.132	59 (52.7)	15.0	0.991
Female	207 (46.8)	25.2		155 (35.1)	32.0		52 (46.4)	14.9	
Unknown	5 (1.13)			4 (1.2)			1 (0.9)		
Age, y									
Mean	66.2			66.5			65.4		
Median	67.0			67.7			67.0		
Range	34–90			37–90			34–90		
T Stage (AJCC 8^th^)									
T1	70 (15.8)	39.0	<0.001	62 (18.8)	48.0	0.003	8 (7.1)	23.0	0.037
T2	262 (59.3)	23.0		190 (57.6)	26.0		72 (64.3)	16.0	
T3	104 (23.5)	15.0		74 (22.4)	19.0		30 (26.8)	9.3	
T4	0 (0.0)	**—**		0 (0.0)	**—**		0 (0.0)	**—**	
Unknown	6 (1.36)			4 (1.2)			2 (1.8)		
N Stage (AJCC 8^th^)									
N0	92 (20.8)	49.0	<0.001	69 (20.9)	54.0	<0.001	23 (20.5)	12.9	0.015
N1	156 (35.3)	26.0		118 (35.8)	35.8		38 (33.9)	17.7	
N2	189 (42.8)	18.0		139 (42.1)	20.0		50 (44.6)	13.0	
Unknown	5 (1.13)			4 (1.2)			1 (0.9)		
Grade/tumor differentiation								
I/well	22 (5.0)	33.0	0.002	22 (6.67)	33.0	0.028	0 (0.0)	**—**	0.682
II/moderate	222 (50.2)	26.5		178 (53.9)	30.0		44 (39.3)	16.0	
III/poor	138 (31.2)	18.0		83 (25.2)	19.0		55 (49.11)	15.0	
IV/undifferentiated	5 (1.1)	13.3		4 (1.2)	10.2		1 (0.9)	13.3	
Unknown	55 (12.4)			43 (13.0)			12 (10.7)		
Margins (R1 <1 mm)									
Clear	117 (26.5)	31.4	<0.001	91 (27.6)	40.0	<0.001	26 (23.2)	16.0	0.374
Involved	164 (37.1)	18.0		120 (36.4)	19.6		44 (39.3)	12.1	
Not available	161 (36.4)			119 (36.1)			42 (37.5)		
Margins (R10mm)									
Clear	251 (56.8)	25.0	<0.001	190 (57.6)	32.0	<0.001	61 (54.5)	16.0	0.324
Involved	111 (25.1)	19.0		80 (24.2)	20.0		31 (27.7)	12.0	
Not available	80 (18.1)			60 (18.2)			20 (17.9)		
Perineural invasion									
Negative	59 (13.3)	33.3	0.013	38 (11.5)	51.7	0.034	21 (18.8)	23.0	0.057
Positive	374 (84.6)	20.3		285 (86.4)	25.0		89 (79.5)	13.0	
Unknown	9 (2.0)			7 (2.1)			2 (1.8)		
Lymphovascular Invasion								
Negative	211 (47.7)	30.0	<0.001	166 (50.3)	37.0	<0.001	45 (40.2)	16.0	0.040
Positive	218 (49.3)	17.2		153 (46.4)	19.0		65 (58.0)	13.6	
Unknown	13 (2.9)			11 (3.3)			2 (1.8)		
Adjuvant Chemotherapy								
No	98 (22.2)	16.5	0.007	67 (20.3)	19.0	0.078	31 (27.7)	13.0	0.129
Yes	244 (55.2)	26.5		193 (58.5	30.1		51 (45.5)	16.0	
Unknown	100 (22.6)			70 (21.2)			30 (26.8)		

### Neoadjuvant and Adjuvant therapy regimens

For patients that received neoadjuvant therapy (NAT), a modified FOLFIRINOX regimen was administered to patients with good performance status (PS) (0). For those patients with poorer PS (1), gemcitabine either as monotherapy or in combination was administered. Dose reductions or delays were instituted at the discretion of the medical oncology team. When given, chemoradia-tion (CRT) with 50.4 Gy and GemCAP were administered.^14^ Following resection, adjuvant therapy was administered if patient performance status allowed it and the regimen was left to the discretion of the treating oncologist based on local and international guidelines. Some patients were enrolled in previous randomized trials (ESPAC trials) and included in previous studies.^5,15–23^

### Pathology Assessment

Patients with resected PDAC were staged according to the AJCC 8^th^ staging criteria, with T-stage based on maximum tumor diameter, and N-stage determined by the number of positive lymph nodes har-vested.^7,12^ Margin involvement (R1) was defined according to the Royal College of Pathologists criteria as the presence of tumor at or < 1mm (R1<1mm) of a margin or surface when assessed by microscopy of a hematoxylin and eosin stained slide. This criterion has proven capable of discriminating outcome following resection.^9–11^ For the APGI and Verona cohorts, margin status was originally defined as evidence of tumor at any margin or surface (R10mm). Where possible, these were re-staged according to R1<1mm with a separate analysis performed for each margin criteria (R1_0mm_ and R1<_1mm_) for these cohorts.

### Transcriptomic Profiling

The molecular subtyping criteria was generated as part of the ICGC landmark study of PDAC.^5^ RNA was extracted from bulk tumor and profiled using RNA sequencing (RNAseq) and gene expression microarrays as previously described.^5^ **Selecting patient samples to** undergo sequencing was based on a number of factors, including cost, tissue quality, and tumor cellularity. All samples were fresh frozen upon collection. Tumors with cellularity <40% *(n* = 249) underwent gene expression micro-array analysis, whereas RNA sequencing was performed in those tumor specimens with cellularity > 40% (n =193) since there is strong evidence to suggest that bulk tumor RNA profiling technologies are comparable.^24^ Individual tumors were classified as either squamous or classical pancreatic subtypes. The classical pancreatic subtype encompassed the pancreatic progenitor, aberrantly differentiated endocrine exocrine (ADEX), and immunogenic subclasses described by Bailey et al.^3^ Tumors underwent molecular subtyping from 4 independent cohorts. The APGI (n = 90) and Verona (n = 103) cohorts underwent RNA sequencing (total n = 193). The remaining patients (n = 249) from the APGI (n = 174), Glasgow (n = 47), and Milan (n = 28) cohorts underwent validated targeted RNA expression and micro-array gene expression analysis^5^. Differential gene expression was performed using the standard pipeline from the Bioconductor [Bioconductor.org] package *“limma”*in groups with positive and negative resection margins. Expression counts were processed and normalized as previously described^5^. S100A2 and S100A4 protein expression were used as surrogate immunohistochem-ical (IHC) biomarkers of the squamous subtype as previously described.^25^ Briefly, high S100A2 expression was defined as cytoplasmic staining with intensity 3+ in >30% of cells and positive S100A4 expression was defined as either nuclear and/or cytoplasmic staining of any intensity in >1% of cells.^25^

### Statistical Analysis

Categorical variables were compared using the chi-square test. The Mann-Whitney *U* test was used to compare continuous variables. The principal outcome measure was length of disease specific survival (DSS) as measured from the time of original surgery, or commencement of NAT. Patients alive at the time of follow-up point were censored. The last follow-up period for patients still alive was October 2020. KaplanMeier survival analysis was used to analyze the DSS. To compare the length of survival between curves, a log-rank test was performed. A Cox proportional hazards model was used for univariate analysis to adjust for competing risk factors, and the hazard ratio (HR) with 95% confidence intervals (CIs) was reported as an estimate of the risk of DSS. Variables found to be significant on univariate analysis at *P <* 0.10 were included in multivariate analysis in a backwards stepwise fashion. Statistical analysis was performed using SPSS (Version 25.0; IBM SPSS Statistics, IBM Corporation, Armonk, NY) and R 3.4.0 (The R Project for Statistical Computing, Vienna, Austria).

## Results

### Patients and Outcomes

Patient demographic, operative, and pathological features are summarized and are consistent with previous published PDAC cohorts^7,10,12^ (Table 1 and supplementary table 1, http://links.lww. com/SLA/D251).

The APGI cohort consisted of 518 patients whom all received upfront surgery. The Glasgow cohort consisted of 366 patients of which 70 (19.1%) received NAT. The RNSH cohort consisted of 283 patients, of which 129 (45.6%) received NAT (supplementary table 1, http://links.lww.com/SLA/D251). The Verona cohort consisted of 103 patients and the Milan cohort consisted of 28 patients of which only 1 patient received neoadjuvant therapy in each cohort. Adjuvant chemotherapy was administered in 206 (39.8%) patients from the APGI cohort, 202 (45.9%) patients from the Glasgow cohort, 218 (77%) patients from the RNSH cohort, 55 (53.4%) in the Verona cohort, and 15 (53.6%) in the Milan cohort.

At the most recent follow-up, 60 (16.4%), 89 (17.2%), 123 (43.6%), 26 (25.2%), and 13 (46.4%) patients were alive for the Glasgow, APGI, RNSH, Verona and Milan cohorts respectively. The median follow-up was 41.0months (range, 3.2–166), 47.0 months (range, 18.0–164), 33.0months (range, 11.1–99), 56.5 months (7.093.0), and 17.5 months (6.0–55.0) for the Glasgow, APGI, RNSH, Verona, and Milan cohorts, respectively (Supplementary Table 1, http://links.lww.com/SLA/D251). The median survival was 25.3 months (18% 5-year DSS), 20.9 months (17% 5-year DSS), 32.7 months (33% 5-year DSS), 28.0 months (29% 5-year DSS) for the RNSH cohort, and 19.0months (42% 5-year DSS) for the Milan cohort.

Of the 1298 patients, 442 (34%) underwent transcriptomic subtyping (molecular subtype cohort) using either RNA sequencing *(n* = 193) or microarray gene expression analysis *(n* = 249) (supplementary figure 1, http://links.lww.com/SLA/D251, supplementary table 1, http://links.lww.com/SLA/D251). Of these n = 330 (74.7%) were classified as classical pancreatic and *n* = 112 (25.3%) as squamous subtype. Significant clinicopathological differences between the 2 subtypes included perineural invasion being more common in the classical subtype (88.3% vs 80.9%, *P* = 0.049), whereas high-grade (58.2% vs 29.8%, *P* < 0.001), lymphovascular invasion (59.1% vs 47.8%, *P* = 0.041) and pancreatic body/tail tumors more frequently in the squamous subtype (as previously described^26^) (supplementary table 2, http://links.lww.com/SLA/D251).

**Figure 1:**
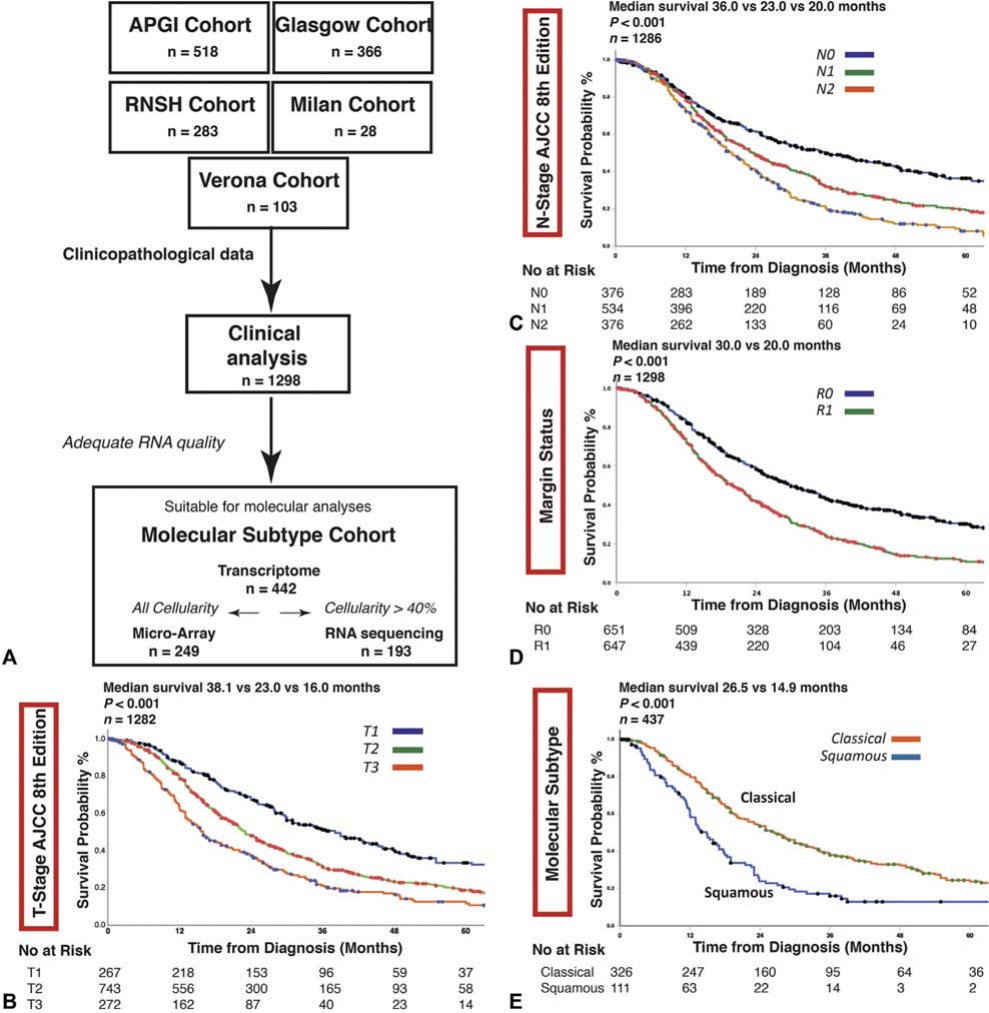
A, Transcriptomic profiling strategy of the molecular subtype cohort. Transcriptomic analysis was performed using either RNA sequencing (RNAseq) or gene expression microarray based on cellularity and adequate RNA quality of the sample in a selection *(n* = 442) of PDACs. Tumor cellularity >40% allowed whole genome sequencing, whilst RNAseq was performed in tumors with sufficient cellularity (>40%) and quality RNA. Kaplan-Meier survival curves for all patients stratified by AJCC 8^th^ edition staging criteria for B) T-stage, C) N-stage, and D) margin status and E) molecular subtype.

**Table 2:** Molecular Subtype Cohort Multivariate Analysis (Final Model)

	Multivariate Analysis	
	**HR (95% CI)**	***P***
Lymph node—N1	1.21 (0.73–2.01)	0.47
N2	1.68 (0.98–2.86)	0.058
Grade (high-grade)	1.66 (1.15–2.42)	0.007
Lymphovascular invasion	1.85 (1.28–2.68)	0.001
Tumor location (body/tail)	1.99 (1.24–3.22)	0.005
Adjuvant therapy	0.63 (0.44–0.90)	0.010
Margin (R1 = 1 mm)	1.50 (1.01–2.21)	0.044
Molecular subtype (squamous)	1.54 (1.04–2.28)	0.032

### Validation of AJCC 8^th^ System

The AJCC 8^th^ staging criteria [T-stage (*P* < 0.001), N-stage (*P* < 0.001)] discriminated clearly between prognostic groups in the entire and individual cohorts (Fig. 1, Supplementary figure 1, http://links.lww.com/SLA/D251). Resection margin status was prognostic in the entire cohort (median survival 30.0 vs 20.0 months, *P <* 0.001) (Fig. 1, supplementary figure 1, http:// links.lww.com/SLA/D251). To more closely assess the impact of margin involvement, the combined cohort was stratified by R status, demonstrating prognostic value of the AJCC 8^th^ system in both R0 and R1 groups (supplementary figure 2, http://links. lww.com/SLA/D251).

**Figure 2:**
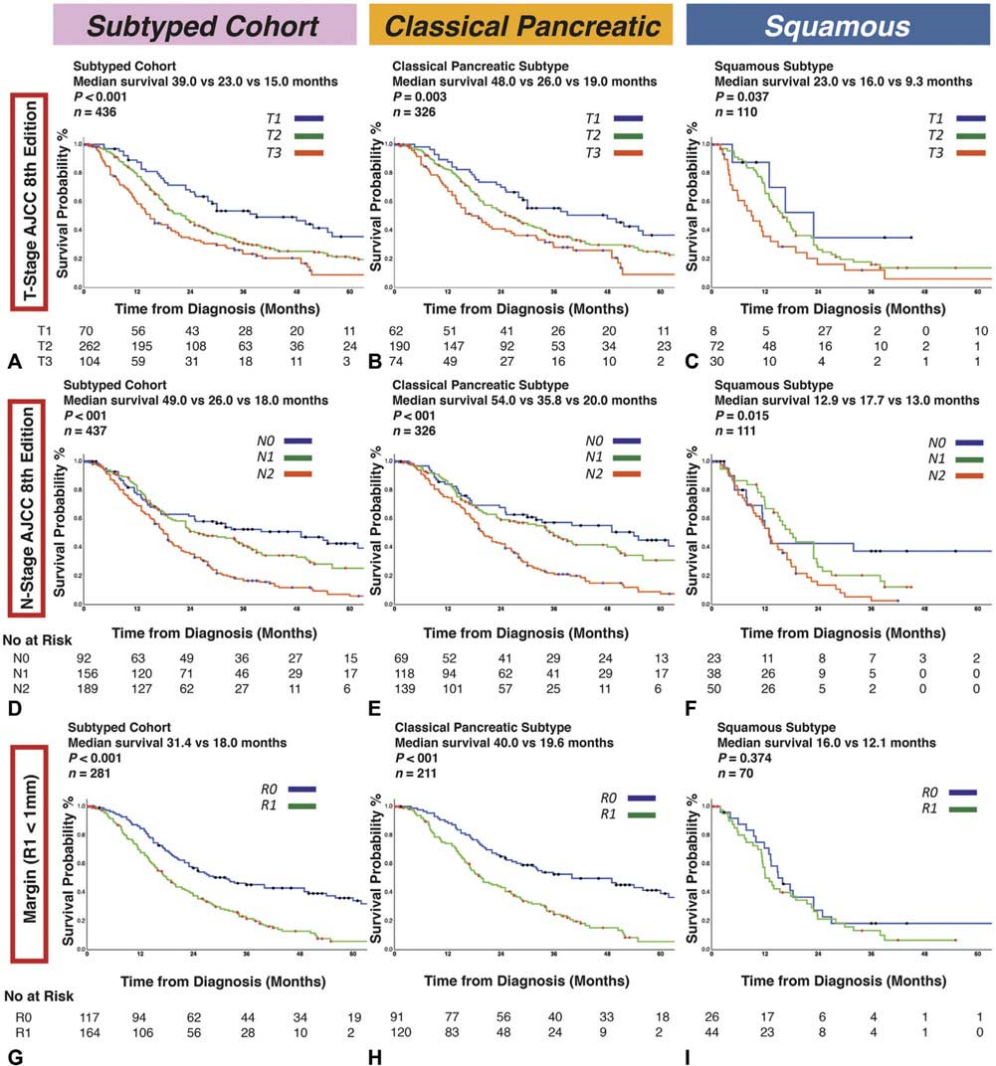
Kaplan-Meier survival curves for the APGI molecular subtype cohort using the AJCC 8^th^ staging criteria. Molecular subtypes were defined according to the Bailey classification as either squamous or classical pancreatic. T-stage is prognostic in (A) the entire cohort, or when stratified according to subtype as (B) classical pancreatic or (C) squamous. N-stage is prognostic in the (D) entire cohort and (E) the classical pancreatic subtype, but not (F) in the squamous subtype. Positive resection margin (R1 ≤1mm) is prognostic in (G) the entire cohort and (H) the classical pancreatic subtype, but not in (I) the squamous subtype.

### Predicting Outcome According to Molecular Subtype

The squamous subtype was associated with significantly worse DSS (median survival 14.9 vs 26.5 months, *P <* 0.001) (Fig. 1). In the molecular subtype cohort (n = 442), T-stage (median survival 39.0 vs 22.0 vs 15.0 months, *P <* 0.001), N-stage (49.0 vs 26.0 vs 18.0months, *P <* 0.001), and R1<_1mm_ status (31.4 vs 18.0 months, *P <* 0.001) predicted DSS (Fig. 2). Squamous subtype associated with poor prognosis in both margin negative (44.0 vs 16.0 months, *P <* 0.001) and margin positive (19.6 vs 12.1 months, *P* = 0.018) cases (supplementary figure 3, http://links.lww.com/SLA/ D251). In this cohort, standard clinical staging criteria T-stage [HR, 2.29 (95% CI, 1.56–3.35); *P* < 0.001 for T3], N-stage [HR, 2.34 (95% CI, 1.89–3.26]; *P* < 0.001 for N2), and R1<_1mm_ [HR, 2.03 (95% CI, 1.51–2.73); *P* < 0.001] were prognostic in univariate analysis (supplementary table 3, http://links.lww.com/SLA/D251). On multivariate analysis, squamous molecular subtype [HR, 1.54 (95% CI, 1.042.28), *P* = 0.032], along with tumor grade [HR, 1.66 (95% CI, 1.15–2.42); *P* = 0.001], lymphovascular invasion [HR, 1.85 (95% CI, 1.28-2.68); *P* = 0.001], tumor location in the body/ tail [HR, 1.99 (1.24–3.22); *P* = 0.005], adjuvant therapy [HR, 0.63 (0.44-0.90); *P* =0.010] and margin (R1<_1mm_) status [HR, 1.50 (95% CI, 1.01–2.21); *P* = 0.044] remained independent predictors of poor prognosis (Table 2, supplementary table 4, http://links.lww.com/ SLA/D251).

**Figure 3:**
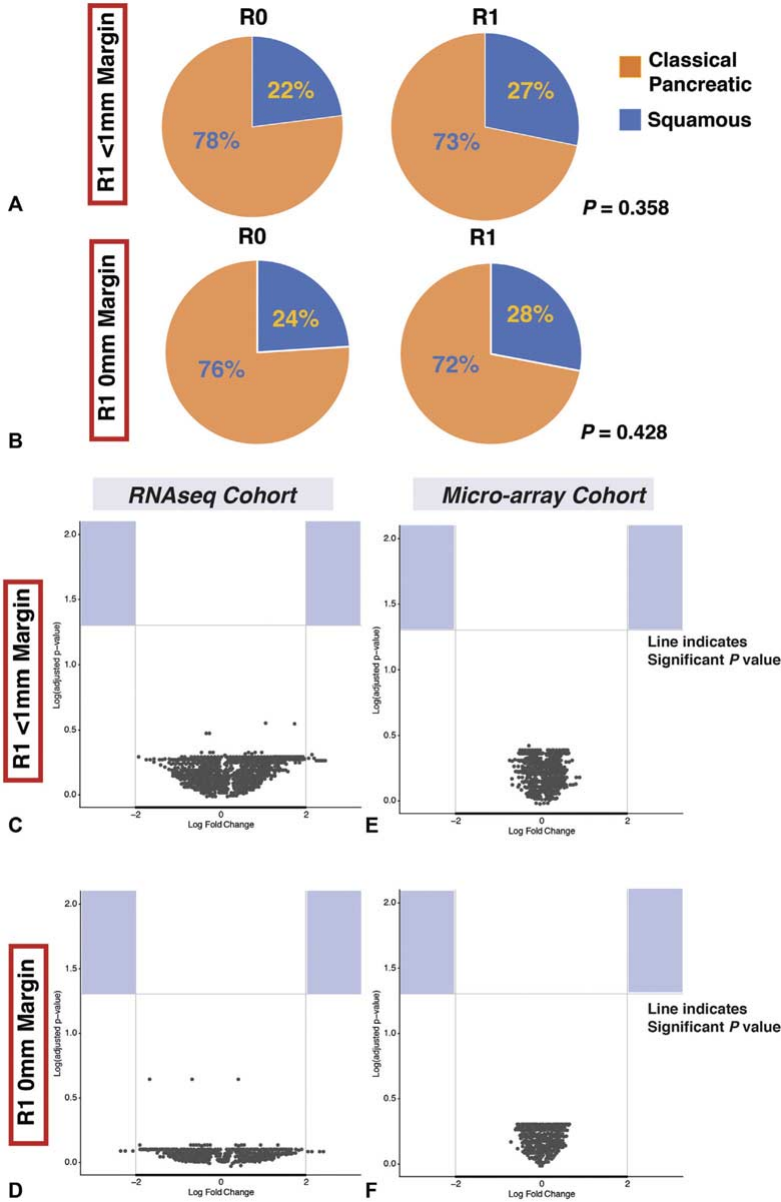
Molecular differences between margin positive and negative PDAC. There was no significant difference between molecular subtype (A, B) or in gene expression using both RNAseq (C, E) or microarray analysis (D, F) between margin positive and margin negative PDAC. Significantly enriched genes are deemed up or downregulated at threshold of −2or +2. Log adjusted P value using Benjamini and Hochberg method. None of the up or downregulated genes reached this significance threshold.

When stratifying patients by molecular subtype, T-stage (median survival 48.0 vs 26.0 vs 19.0months, *P* = 0.003), N-stage (54.0 vs 35.8 vs 20.0 months, *P* < 0.001), and R1<_1mm_ status (40.0 vs 19.6 months, *P* < 0.001) predicted DSS in the classical pancreatic subtype (Fig. 2). For patients with the squamous subtype, however, N-stage (12.9 vs 17.7 vs 13.0months, *P* = 0.015) and R1 status [both R1≤1mm (16.0 vs 12.1 months, *P* = 0.374) (Fig. 2, supplementary figure 4, http://links.lww.com/SLA/D251), and R10mm (16.0 vs 12.0 months, *P* = 0.324) criteria (supplementary figure 4, http:// links.lww.com/SLA/D251)] did not predict DSS.

The classical subtype was assessed individually with high tumor grade [HR, 2.26 (1.44–3.54); *P* < 0.001], margin (R1<_1mm_) status [HR, 2.10 (1.40–3.17); *P* < 0.001], lymphovascular invasion [HR, 2.46 (1.62–3.73); *P* <0.001], T-stage [HR, 2.23 (1.14–4.35); *P* = 0.019] and adjuvant chemotherapy [HR, 0.62 (0.40–0.95); *P* = 0.028] being independent predictors of DSS (supplementary table 5, http://links.lww.com/SLA/D251). When the squamous subtype was assessed, only tumor location in the body/tail [HR, 2.46 (1.17–5.17); *P* = 0.018] was independently associated with DSS (supplementary table 6, http://links.lww.com/SLA/D251), demonstrating the differential impact of staging factors and margin status on prognosis in different molecular subtypes. The squamous subtype was less likely to receive adjuvant therapy (supplementary table 7, http://link-s.lww.com/SLA/D251) and had a significantly worse prognosis, irrespective of whether adjuvant therapy was administered or not (supplementary figure 5, http://links.lww.com/SLA/D251). Only n = 16 patients underwent neoadjuvant therapy, with a variety of regimens used (supplementary table 8, http://links.lww.com/SLA/D251) and the impact of this on subtype could not be assessed.

The impact of S100A2 and S100A4 protein expression on pathological staging was investigated in patients from the molecular subtype cohort that had immunohistochemistry data available to assess the utility of simple IHC biomarkers of the squamous sub-type^25^. S100A2and S100A4 expression did not have the same impact on margin and nodal status as full molecular subtyping using gene expression (supplementary figure 6, http://links.lww.com/SLA/ D251), with both margin and lymph node status remaining prognostic despite expression of both biomarkers.

### Molecular Features of Margin-Positive PDAC

Since molecular features are crucial to prognosis, even in the setting of a positive resection margin (supplementary figure 3, http:// links.lww.com/SLA/D251) we investigated the molecular differences in margin positive and negative PDAC. Resection margin status, both at R1_0mm_ or R1 _<1mm_, was not associated with molecular subtype (Fig. 3). Differential gene expression analysis from the APGI molecular subtype cohort demonstrated no discernible difference between tumors defined as resection margin positive or negative (Fig. 3). RNAseq and gene expression microarray analysis demonstrated no significant differences in gene expression between the groups, even when R1_0mm_ status was used.

## Discussion

Comprehensive characterization of pancreatic cancer resection specimens should include evaluation of molecular profile. In 1298 resected PDACs we validated the prognostic value of the AJCC 8^th^ staging system incorporating detailed margin status annotation. Yet stratification according to molecular subtype (aggressive disease biology), confounded the prognostic value of this pathological staging system, and negated the impact of resection margin involvement. This suggests that biological factors including molecular subtype convey significant prognostic value with potential to impact the personalization of surgical management algorithms.

The AJCC 8^th^ staging criteria has been validated in multiple unselected cohorts of resected PDAC, significantly improving prognostication for patients.^7,12^ This is particularly pronounced with the updated N-stage criteria discriminating according to lymph node metastases burden, however, modifications have already been proposed to further enhance outcome prediction.^27^ These studies remain limited, as there has been a failure to account for heterogeneity driven by tumor biology and molecular determinants of disease outcome.

Transcriptomic subtyping has transformed our understanding of the molecular taxonomy for most cancers.^3^ The existence of two distinct PDAC subtypes has been demonstrated and validated in numerous classifiers, suggesting the concept of opposing lineages is robust.^3,5,28–33^ Previous studies have demonstrated the squamous molecular subtype to be associated with poor prognosis, whereas a simplified protein-based expression of a squamous biomarker predicts poor outcome following upfront pancreatectomy.^4,5^ No previous study has investigated the impact of molecular subtype on pathological staging criteria such as margin or lymph node status, which makes the results presented in this study novel.

Patients with the squamous molecular subtype had a significantly reduced median DSS compared to patients with the classical subtype. We have demonstrated that molecular transcriptomic analysis using a variety of techniques can robustly subtype patients from independent institutions following resection of PDAC. Resection margin status, particularly R1_<1mm,_ is a critical independent predictor of outcome following pancreatectomy for PDAC, including in prospective clinical trial analysis ^9,10,34^. Our results suggest that for patients with the aggressive squamous molecular phenotype, both lymph node involvement and R1 status fail to impact prognosis. Presumed metastatic dissemination, occurring early in pancreatic carcinogenesis, driven by particular molecular features in the squa-mous subtype may explain this finding. Although the mechanisms require elucidation, these results have significant implication for clinical trial design and interpretation particularly if R1 status is employed as a surrogate endpoint. The current results confer support for the squamous molecular subtype being regarded as a disease entity distinct from the classical pancreatic subtype, particularly in combination with preliminary evidence that suggests chemothera-peutic response differs according to transcriptomic subtype.^35^

It appears that the classical pancreatic subtype is the default molecular lineage with evolution into squamous subtype occurring in some patients.^32,36^ Previous work from our group suggests that the squamous molecular subtype is more frequent in PDAC originating within the pancreatic body and tail.^26^ Whether this is determined early in carcinogenesis or if the squamous subtype simply reflects molecular evolution requires elucidation. Interestingly, the classical pancreatic subtype had a higher frequency of perineural invasion (88% vs 81%, *P* = 0.049). The reason for this is not clear from this study but may be that classical tumors are more likely to invade locally and cause local recurrence. Whereas squamous tumors are more likely to cause early distant, hepatic recurrence and may explain its association with lymphovascular invasion (59% vs 47%). In this study, we used bulk tumor samples for gene expression analysis and subtyping. Recently, several studies have demonstrated that molecular subtype can exist on a spectrum within the same tumor.^32,36^ Bulk tumor transcriptomic sequencing likely classify tumors based on the dominant subtype, yet further study is required to investigate the extent of transcriptomic subtype intra-tumoral heterogeneity and how this impacts clinical outcome.^36^

Our results demonstrated no difference in the gene expression of tumors deemed margin positive versus those that were margin negative. Resection margin status is often viewed as a surrogate of aggressive tumor biology; however, in this cohort, there were no significant transcriptomic differences. This may result from margin status being influenced by other biological factors, for example, tumor microenvironment composition or immune infiltration. Spatial transcriptomic characterization has the potential to elucidate subtype heterogeneity and impact of the microenvironment on margin status.^36,37^ Ultimately, margin status, may simply reflect anatomical location of the tumor, rather than a surrogate for tumor biology. This is particularly apparent for the squamous subtype, where local control by clear margins appears to be less crucial due to early systemic dissemination in these patients. There is some evidence that margin involvement in proximity to vasculature or neural plexus is associated with local recurrence.^10^ Prospective studies, with comprehensive molecular and pathological characterization, are necessary to further elucidate this.

Based on results presented here, and by others^4,5^, we support the concept that comprehensive staging for a patient diagnosed with PDAC, particularly in the potentially operable setting [resectable, borderline resectable (BR) and locally advanced (LA)], should integrate biological predictors of disease prognosis.^38^ To date, attempts have been made to identify aggressive tumor biology utilizing tumor markers (CA19–9) or tumor stage, without accounting for the molecular features that make up and drive each individual tumor.^39^ Molecular characterization of the patient and tumor, both at the transcriptomic and genomic levels, at the time of diagnostic biopsy may enable better selection of patients for resection, optimizing high-risk surgical management strategies for patients with BR and LA PDAC.^4^

The natural progression of health care, and cancer treatment, trends toward a precision medicine strategy where therapy selection aligns with individual and tumor features. Despite development of novel personalized molecular and histological tools that evaluate tumor biology,^4^ these metrics have as yet failed to integrate into clinical practice. For PDAC, the evolution toward a precision oncology strategy is driven by global initiatives including *PRECISION-Panc* in the United Kingdom, which aim to harness molecular variation to guide therapy. This study suggests that using only pathological staging as a predictor of postoperative outcome fails to encompass the biological attributes of the tumor. This, in turn will impact the utility of clinical and pathological staging, particularly in the setting of clinical trials where survival or margin status is often used as study endpoints. We propose that molecular subtyping is determined in these settings to accurately compare new treatment regimens and avoid unaccounted biases in clinical studies and prospective trials.

This study is limited firstly as the majority of molecularly subtyped tumors were not treated with NAT, an increasingly common strategy for managing PDAC, and thus the impact of response to NAT is not considered. A previous study has suggested that neoadjuvant FOLFIRINOX may induce evolution from the classical to the squa-mous subtype,^40^ yet we were unable to investigate this here. There is growing evidence that the squamous (or basal) subtype is less likely to respond to current chemotherapeutic regimes than Classical Pancreatic subtype.^35^ In this cohort, the squamous subtype were less likely to receive adjuvant chemotherapy which the authors believe demonstrates likely significant micro metastatic disease burden leading to poor performance status post pancreatectomy, which in turn impedes adjuvant therapy allocation. Circulating biomarkers such as CA19–9 and circulating tumor cells (CTCs) were not available for a large proportion and thus could not be assessed. The relationship between molecular features, therapeutic response and prognosis is complex and remains to be determined. The PRIMUS–002 neoadjuvant trial (ISRCTN34129115), part of the *PRECISION-Panc* platform (ISRCTN14879538), is currently recruiting, and aims to delineate the interaction of genomic and transcriptomic molecular subtype, circulating biomarkers (CA19-9, CTCs and cell free DNA), therapeutic response and prognosis in the setting of non-metastatic PDAC.^6^ This prospective trial will provide molecular subtype characterization based on preoperative biopsies and resection specimens, which will allow investigation of the impact of neoadjuvant therapy on molecular subtype evolution and heterogeneity.

## Conclusions

These results demonstrate that biological characteristics as determined by transcriptomic subtype are a strong predictor of outcome following pancreatectomy forPDAC. Standard pathological staging criteria, particularly margin status, in PDAC failed to predict outcome in patients with tumors of the squamous molecular subtype. This indicates that tumor biology should be accounted for when staging patients following surgical resection of PDAC, particularly in the setting of clinical trials, as these features have potential to personalize both treatment allocation and prognosis. We envisage soon that transcriptomic subtype, in addition to genomic characterization, will be determined preoperatively and facilitate patient centered algorithms to improve outcomes for patients with PDAC.

## Supplementary Material
