# Supplementary material for: The Impact of Molecular Subtyping on Pathological Staging of Pancreatic Cancer
Source: Ann Surg. 2023 Jan 10;277(2):e396–405. doi: 10.1097/SLA.0000000000005050 (PMC9831035; doi:10.1097/SLA.0000000000005050)
Supplement: Supplementary file 1 [file sla-277-e396-s001.pdf]

Supplementary File:

The impact of molecular subtyping on pathological staging of Pancreatic Cancer

Stephan B Dreyer[1,2] MD, PhD, Sarah Rae[1] BMSc, Kirsty Bisset[2] MD, Rosie Upstill-Goddard[1] PhD, Georgios Gemenetzis[2] MD, Amber L. Johns[3] BSc, Australian Pancreatic Cancer Genome Initiative[3], Glasgow Precision Oncology Laboratory[1], Euan J. Dickson[2] MD, Anubhav Mittal[4, 5] MD PhD, Anthony J. Gill[3,6,7] MD, PhD, Fraser Duthie[1, 8] MD, PhD, Antonio Pea[1,9] MD, PhD, Rita T. Lawlor[10] PhD, Aldo Scarpa[10,11] MD, PhD, Roberto Salvia[9] MD, Alessandra Pulvirenti[12] MD, Alessandro Zerbi[12,13] MD, Federica Marchesi[14, 15] PhD, , Colin J. McKay[1,2] MD, Andrew V. Biankin[1,2] MD, PhD, Jaswinder S. Samra[4, 5] MD, David K. Chang*[1,2] MD, PhD and Nigel B Jamieson*[1,2,4] MD, PhD

[1]Wolfson Wohl Cancer Research Centre, Institute of Cancer Sciences, University of Glasgow, Garscube Estate, Switchback Road, Bearsden, Glasgow, Scotland G61 1QH, United Kingdom ; [2]West of Scotland Pancreatic Unit, Glasgow Royal Infirmary, Glasgow G31 2ER UNITED KINGDOM; [3]The Kinghorn Cancer Centre, 370 Victoria Street, Darlinghurst and Garvan Institute of Medical Research, Sydney, NSW 2010, AUSTRALIA; [4]Department of Surgery, Royal North Shore Hospital, St Leonards, Sydney NSW 2065, Australia; [5]University of Sydney, Sydney, NSW 2006, Australia; [6]Cancer Diagnosis and Pathology Group Kolling Institute of Medical Research and Department of Anatomical Pathology, Royal North Shore Hospital. Sydney NSW 2065, AUSTRALIA; [7]Northern Clinical School, Faculty of Medicine, University of Sydney, AUSTRALIA; [8]Department of Pathology, Queen Elizabeth University Hospital, Glasgow, G51 4TF, UNITED KINGDOM; [9] General and Pancreatic Surgery Department, Pancreas Institute, University and Hospital Trust of Verona, Verona, ITALY; [10]ARC-Net Research Centre, University and Hospital Trust of Verona, Verona, ITALY; [11]Department of Diagnostics and Public Health, University and Hospital Trust of Verona, Verona, ITALY. [12]Pancreatic Surgery Unit, IRCCS Humanitas Research Hospital, Rozzano, Milan, ITALY; [13]Department of Biomedical Sciences, Humanitas University, Pieve Emanule, Milan, ITALY; [14]Department of Immunology, IRCCS Humanitas Research Hospital, Rozzano, ITALY; [15]Department of Medical Biotechnology and Translational Medicine, University of Milan, Milan, ITALY.

Research Funding Support:

- Wellcome Trust Senior Investigator Award: 103721/Z/14/Z
- Cancer Research UK Programme: C29717/A17263 and C29717/A18484
- Cancer Research UK Glasgow Centre: C596/A18076
- Cancer Research UK Clinical Training Award: C596/A20921
- Cancer Research UK Clinician Scientist Award: C55370/A25813
- The National Health & Medical Research Council of Australia, The Cancer Council NSW, Cancer Institute NSW, Royal Australasian College of Surgeons, St Vincent's Clinic Foundation and R. T. Hall Trust.
- The Australian Pancreatic Cancer Foundation
- Associazione Italiana Ricerca Cancro (AIRC 5x1000 n. 12182);
- Fondazione Italiana Malattie Pancreas – Ministero Salute [FIMPCUP_J38D19000690001]
- Fondazione Cariverona: Oncology Biobank Project "Antonio Schiavi" (prot. 203885/2017)
- IG2020-24393-Associazione Italiana per la Ricerca su Cancro

Corresponding authors:
Nigel B Jamieson
Wolfson Wohl Cancer Research Centre,
Institute of Cancer Sciences, University of Glasgow,
Garscube Estate, Switchback Road,
Bearsden, Glasgow Scotland G61 1BD
Tel: +44 141 330 5670 Fax: +44 141 330 5834
Email: nigel.jamieson@glasgow.ac.uk

David K. Chang
Wolfson Wohl Cancer Research Centre,
Institute of Cancer Sciences, University of Glasgow,
Garscube Estate, Switchback Road,
Bearsden, Glasgow Scotland G61 1BD
Tel: +44 141 330 7589 Fax: +44 141 330 5834
Email: david.chang@glasgow.ac.uk

Running Head: Staging Pancreatic Cancer in era of molecular subtyping

Keywords: Pancreatic Cancer, Molecular Subtyping, Cancer Staging

Conflicts of Interest: There are no conflicts of interests to declare in relevance to this piece of work

Australian Pancreatic Cancer Genome Initiative

Garvan Institute of Medical Research Amber L. Johns[1], Anthony J Gill[1, 5], Lorraine A. Chantrill[1,22], Paul Timpson[1,] Angela Chou[1,5], Marina Pajic[1], Tanya Dwarte[1], David Hermann[1], Claire Vennin[1], Thomas R Cox[1], Brooke Pereira[1], Shona Ritchie[1], Daniel A Reed[1], Cecilia R Chambers[1], Xanthe Metcalf[1], Max Nobis[1], **QIMR Berghofer Medical Research Institute** Nicola Waddell[2], John V. Pearson[2], Ann-Marie Patch[2], Katia Nones[2], Felicity Newell[2], Pamela Mukhopadhyay[2], Venkateswar Addala[2], Stephen Kazakoff[2], Oliver Holmes[2], Conrad Leonard[2], Scott Wood[2]. **University of Melbourne, Centre for Cancer Research** Sean M. Grimmond[3], Oliver Hofmann[3]. **Royal North Shore Hospital** Jaswinder S. Samra[5], Nick Pavlakis[5,] Jennifer Arena[5], Hilda A. High[5]. **Bankstown Hospital** Ray Asghari[6], Neil D. Merrett[6], Amitabha Das[6]. **Liverpool Hospital** Peter H. Cosman[7], Kasim Ismail[7]. **St Vincent's Hospital** Alina Stoita[8], David Williams[8], Allan Spigellman[8]. **Westmead Hospital** Vincent W. Lam[9,] Duncan McLeod[9], Judy Kirk[9]. **Royal Prince Alfred Hospital, Chris O'Brien Lifehouse** James G. Kench[10], Peter Grimison[10], Charbel Sandroussi[10], Annabel Goodwin[7,10]. **Prince of Wales Hospital** R. Scott Mead[1,11], Katherine Tucker[11], Lesley Andrews[11]. **Fiona Stanley Hospital** Michael Texler[12], Cindy Forrest[12], Mo Ballal[12,13], David Fletcher[12]. **St John of God Healthcare** Maria Beilin[13], Kynan Feeney[13] Krishna Epari[13] Sanjay Mukhedkar[13]. **Epworth HealthCare** Nikolajs Zeps[23]. **Royal Adelaide Hospital** Nan Q Nguyen[14], Andrew R. Ruszkiewicz[14], Chris Worthley[14]. **Flinders Medical Centre** John Chen[15], Mark E. Brooke-Smith[15], Virginia Papangelis[15]. **Envoi Pathology** Andrew D. Clouston[16]. **Princess Alexandria Hospital** Andrew P. Barbour[17], Thomas J. O'Rourke[17], Jonathan W. Fawcett[17], Kellee Slater[17], Michael Hatzifotis[17], Peter Hodgkinson[17]. **Austin Hospital** Mehrdad Nikfarjam[18]. **Johns Hopkins Medical Institutes** James R. Eshleman[19], Ralph H. Hruban[19], Christopher L. Wolfgang[19]. **ARC-Net Centre for Applied Research on Cancer** Aldo Scarpa[20], Rita T. Lawlor[20], Vincenzo Corbo[20], Claudio Bassi[20]. **University of Glasgow** Andrew V Biankin [21], Nigel B. Jamieson[21] David K. Chang[1, 21,] Stephan B. Dreyer[21].

[1]The Kinghorn Cancer Centre, Garvan Institute of Medical Research, 370 Victoria Street, Darlinghurst, Sydney, New South Wales 2010, Australia.

[2]QIMR Berghofer Medical Research Institute, 300 Herston Rd, Herston, Queensland 4006, Australia.

[3]University of Melbourne, Centre for Cancer Research, Victorian Comprehensive Cancer Centre, 305 Grattan Street, Melbourne, Victoria 3000, Australia.

[4] Institute for Molecular Bioscience, University of QLD, St Lucia, Queensland 4072, Australia.

[5]Royal North Shore Hospital, Westbourne Street, St Leonards, New South Wales 2065, Australia.

[6]Bankstown Hospital, Eldridge Road, Bankstown, New South Wales 2200, Australia.

[7]Liverpool Hospital, Elizabeth Street, Liverpool, New South Wales 2170, Australia.

[8] St Vincent's Hospital, 390 Victoria Street, Darlinghurst, New South Wales, 2010 Australia.

[9]Westmead Hospital, Hawkesbury and Darcy Roads, Westmead, New South Wales 2145, Australia.

[10]Royal Prince Alfred Hospital, Missenden Road, Camperdown, New South Wales 2050, Australia.

[11]Prince of Wales Hospital, Barker Street, Randwick, New South Wales 2031, Australia.

[12]Fremantle Hospital, Alma Street, Fremantle, Western Australia 6959, Australia.

[13] St John of God Healthcare, 12 Salvado Road, Subiaco, Western Australia 6008, Australia.

[14] Royal Adelaide Hospital, North Terrace, Adelaide, South Australia 5000, Australia.

[15] Flinders Medical Centre, Flinders Drive, Bedford Park, South Australia 5042, Australia.

[16] Envoi Pathology, 1/49 Butterfield Street, Herston, Queensland 4006, Australia.

[17] Princess Alexandria Hospital, Cornwall Street & Ipswich Road, Woolloongabba, Queensland 4102, Australia.

[18] Austin Hospital, 145 Studley Road, Heidelberg, Victoria 3084, Australia.

[19] Johns Hopkins Medical Institute, 600 North Wolfe Street, Baltimore, Maryland 21287, USA.

[20] ARC-NET Center for Applied Research on Cancer, University of Verona, Via dell'Artigliere, 19 37129 Verona, Province of Verona, Italy.

[21] Wolfson Wohl Cancer Research Centre, Institute of Cancer Sciences, University of Glasgow, Garscube Estate, Switchback Road, Bearsden, Glasgow, Scotland G61 1BD, United Kingdom.

[22] Wollongong Hospital, Illawarra and Shoalhaven Local Health District, Loftus Street, Wollongong NSW 2500.

[23] Epworth HealthCare, 89 Bridge Rd, Richmond VIC 3121, Australia

Ethics approval numbers

- Sydney Local Health District (RPA Zone) : Approval number X16-0293
- University of Melbourne Health Sciences Human Ethics Subcommittee: Approval Number 1748955.
- North Shore Private Hospital Ethics Committee: Approval number NSPHEC 2016-016.
- Garvan Institute of Medical Research Approval number 1627.

- West of Scotland Research Ethics Service (WoSRES) committee, NHS Greater Glasgow and Clyde-Molecular profiling of pancreatic cancer for improved prediction of Survival. Research Ethics Committee reference number: 07/S0704/26

- Ethics approval for Royal North Shore Hospital cohort was obtained from Northern Sydney Local Health District Human Research and Ethics Committee (NSLHD HREC HREC/16/HAWKE/105).
- Protocol number ICH-595, Humanitas Research Hospital, Milan

All data was transferred under the premise of approved data transfer agreements between the institutions and the University of Glasgow.

Supplementary Tables

Supplementary Table 1: Patient characteristics for the Glasgow, APGI, RNSH and Milan Cohorts															
	Glasgow Cohort			APGI Cohort			Royal North Shore Cohort			Verona Cohort			Milan Cohort		
Variables	n = 366 No. (%)	Median DSS (months)	*P* value (Log-rank)	n = 518 No. (%)	Median DSS (months)	*P* value (Log-rank)	n = 283 No. (%)	Median DSS (months)	*P* value (Log-rank)	n = 103 No. (%)	Median DSS (months)	*P* value (Log-rank)	n = 28 No. (%)	Median DSS (months)	*P* value (Log-rank)
Sex															
Male	192 (52.5)	23.7	0.339	258 (49.8)	18.7		151 (53.4)	27.7	0.677	49 (47.6)	28.0	0.798	12 (42.8)	25.0	0.189
Female	174 (47.5)	25.7		260 (50.2)	21.0	0.573	132 (46.6)	34.1		54 (52.4)	30.0		16 (57.2)	12.0	
Age (years)															
Mean	63.0			66.7			67.1			66.01			70.32		
Median	64.3			68.0			68.4			68.00			72.50		
Range	30.8 – 86.0			28.0 – 88.0			33.9 - 91.3			37 - 87			43 – 85		
Outcome															
Follow-up (months)	3.2 – 166.1			18.0–163.8			11.1 – 98.9			7.0 – 93.0			6 – 55		
Median follow up(months)	41.0			47.0			33.0			56.5			17.5		
Death PDAC	261			394 (76.1)			147			64			15 (53.6)		
Death other	45			31 (6.0)			12			13			0 (0.0)		
Death Unknown	-			3 (0.6)			-			-			0 (0.0)		
Alive	60			89 (17.2)			123			26			13 (46.4)		
Lost to FU	-			1 (0.1)			-			0			0 (0.0)		
Stage (AJCC 8th)															
CPR*	5 (1.4)	NR	< 0.001	-		<0.001	6 (2.1)	NR	<0.001	0 (0.0)	-	0.033	0 (0.0)	N/A	0.150
IA	41 (11.2)	47.7		38 (7.3)	30.0		25 (8.8)	NR		4 (3.9)	55.0		1 (3.6)		
IB	36 (9.8)	26.7		94 (18.1)	19.6		46 (16.3)	34.9		7 (6.8)	32.0		4 (14.3)		
IIA	19 (5.2)	31.0		36 (6.9)	13.0		7 (2.5)	78.6		3 (2.9)	NR		2 (7.1)		
IIB	149 (40.7)	19.0		227 (43.8)	20.4		116 (41.0)	33.1		33 (32.0)	51.0		8 (28.6)		
III	116 (31.7)	22.4		108 (20.8)	17.0		83 (29.3)	23.9		56 (54.4)	22.0		13 (46.4)		
T Stage (AJCC 8th)															
CPR*	5 (1.4)	NR					6 (2.1)	NR		0 (0.0)	-		0 (0.0)		
T1	81 (22.1)	39,0	<0.001	101 (19.5)	34.4	<0.001	48 (17.0)	NR	0.011	33 (32.0)	30.0	0.358	4 (14.3)	NR	0.511
T2	207 (56.6)	22.6		292 (56.4)	18.6		175 (61.8)	30.5		56 (54.4)	28.0		13 (46.4)	14.0	
T3	73 (19.9)	13.5		119 (23.0)	13.2		53 (18.7)	27.6		14 (13.6)	14.0		11 (39.3)	19.0	
T4	0 (0.0)	-		2 (0.3)	-		1 (0.4)	-		0 (0.0)	-		0 (0.0)	-	
Unknown	0 (0.0)			4 (0.8)			0 (0.0)			0 (0.0)	-		0 (0.0)	-	
N Stage (AJCC 8th)															
N0	101 (27.6)	47.7	<0.001	169 (32.6)	22.2	<0.001	85 (30.0)	61.1	<0.001	14 (13.6)	55.0	0.007	7 (25.0)	NR	0.041
N1	149 (40.7)	19.0		228 (44.0)	20.3		116 (41.0)	33.1		33 (32.0)	51.0		8 (28.6)	NR	
N2	116 (31.7)	22.4		109 (21.0)	16.0		82 (29.0)	23.9		56 (54.4)	22.0		13 (46.4)	13.0	
Unknown	0 (0.0)			12 (2.3)			0 (0.0)			0 (0.0)			0 (0.0)		
Tumor Differentiation															
I / Well	30 (8.2)	36.3	<0.001	41 (7.9)	35.4	0.039	14 (4.9)	46.4	0.059	5 (4.8)	30.0	0.480	1(3.6)	NR	0.271
II / Moderate	229 (62.6)	25.0		340 (65.6)	21.0		186 (65.7)	34.1		62 (60.2)	30.0		6 (21.4)	NR	
III / Poor	100 (27.3)	17.1		130 (25.1)	17.0		76 (26.9)	26.5		35 (34.0)	19.0		21 (75.0)	17.0	
IV / undifferentiated	0 (0.0)	-		5 (1.0)	13.0		0 (0.0)	-		0 (0.0)	-		0 (0.0)		
Unknown	7 (1.9)	-		2 (0.4)			7 (2.5)			1 (1.0)	-		0 (0.0)		
Resection margin†															
Clear	119 (32.5)	36.7	<0.001	338 (65.3)	23.7	<0.001	123 (43.5)	54.2	0.001	59 (57.3)	48.0	0.002	12 (42.9)	NR	0.134
Involved	247 (67.5)	20.1		180 (15.4)	15.4		160 (56.5)	26.2		44 (42.7)	22.0		16 (57.1)	14.0	
Tumor Location															
Head / Uncinate	328 (89.6)	23.0	0.004	426 (82.2)	22.0	<0.001	268 (94.7)	30.2	0.055	87 (84.5)	29.0	0.848			
Body / Tail	38 (10.4)	39.0		92 (17.8)	12.1		15 (5.3)	40.2		16 (15.5)	23.0				

*CPR = complete pathological response; † = Margin status defined as original pathology report for each cohort (see methods)

Supplementary Table 2: The association of clinicopathological features and molecular subtype

	Classical Pancreatic *n* = 330 (%)	Squamous *n* = 112 (%)	*P*-value (Chi-square)
T - stage			
T1	63 (19.1)	8 (7.3%)	0.013
T2	192 (58.4)	72 (65.5%)	
T3	74 (22.5)	30 (27.3%)	
N - stage			
N0	69 (21.0)	23 (20.7)	0.860
N1	121 (36.8)	38 (34.2)	
N2	139 (42.2)	50 (45.0)	
Tumor location			
Head	276 (88.7)	79 (76.7)	0.002
Body / Tail	35 (11.3)	24 (23.3)	
Grade / Differentiation			
I / II	230 (70.1)	47 (42.7)	<0.001
III / IV	98 (29.9)	63 (57.3)	
Perineural Invasion			
Negative	38 (11.7)	21 (19.1)	0.049
Positive	288 (88.3)	89 (80.9)	
Lymphovascular invasion			
Negative	168 (52.2)	45 (40.9)	0.041
Positive	154 (47.8)	65 (59.1)	
Margin R1 = 1mm			
Negative	92 (43.4)	26 (37.1)	0.358
Positive	120 (56.6)	44 (62.9)	
Margin R1 = 0mm			
Negative	193 (70.7)	61 (66.3)	0.428
Positive	80 (29.3)	31 (33.7)	

Supplementary Table 3: Molecular subtype cohort univariate survival analysis		
	Univariate Analysis	
	HR (95% CI)	*P*
Tumour Grade (High vs low)	1.57 (1.24 – 1.99)	<0.001
Margin Involvement ($R1_{<1mm}$)	2.03 (1.51 – 2.73)	<0.001
Lymphovascular Invasion (Positive)	1.88 (1.49 – 2.37)	<0.001
Perineural Invasion (Positive)	1.56 (1.09 – 2.22)	0.014
Lymph Node stage 8th N1	1.36 (0.96 – 1.92)	0.082
Lymph Node stage 8th N2	2.34 (1.89 – 3.26)	<0.001
T stage (8th edition) T2	1.54 (1.09 – 2.17)	0.013
T stage (8th edition) T3	2.29 (1.56 – 3.35)	<0.001
Adjuvant Chemotherapy	0.68 (0.51 – 0.90)	0.008
Tumor Location (Body/Tail vs Head)	1.45 (1.05 – 1.99)	0.023
Molecular Subtype (Squamous vs Classical)	1.98 (1.54 – 2.56)	<0.001

Supplementary Table 4: Molecular subtype cohort multivariate survival analysis		
	Multivariate Analysis	
	HR (95% CI)	*P*
Step 1		
T stage – T2	1.14 (0.62 – 2.11)	0.676
T3	1.58 (0.81 – 3.08)	0.179
Lymph Node – N1	1.14 (0.68 – 1.90)	0.632
N2	1.54 (0.89 – 2.65)	0.121
Grade (High grade)	1.61 (1.10 – 2.35)	0.015
Perineural Invasion	1.07 (0.63 – 1.82)	0.79
Lymphovascular Invasion	1.83 (1.24 – 2.71)	0.003
Tumor Location (body/tail)	1.83 (1.12 – 2.97)	0.015
Adjuvant Therapy	0.63 (0.44 – 0.90)	0.012
Margin (R1 = 1mm)	1.45 (0.96 – 2.20)	0.076
Molecular Subtype (Squamous)	1.50 (1.00 – 2.25)	0.051
STEP 2		
T stage – T2	1.14 (0.62 – 2.12)	0.672
T3	1.59 (0.81 – 3.09)	0.176
Lymph Node – N1	1.13 (0.68 – 1.90)	0.637
N2	1.54 (0.90 – 2.66)	0.119
Grade (High grade)	1.61 (1.10 – 2.35)	0.015
Lymphovascular Invasion	1.86 (1.29 – 2.69)	0.001
Tumor Location (body/tail)	1.83 (1.13 – 2.98)	0.015
Adjuvant Therapy	0.63 (0.44 – 0.91)	0.013
Margin (R1 = 1mm)	1.48 (1.00 – 2.19)	0.049
Molecular Subtype (Squamous)	1.49 (1.00 – 2.22)	0.053
Step 3 – Final Model		
Lymph Node – N1	1.21 (0.73 – 2.01)	0.47
N2	1.68 (0.98 – 2.86)	0.058
Grade (High grade)	1.66 (1.15 – 2.42)	0.007
Lymphovascular Invasion	1.85 (1.28 – 2.68)	0.001
Tumor Location (body/tail)	1.99 (1.24 – 3.22)	0.005
Adjuvant Therapy	0.63 (0.44 – 0.90)	0.010
Margin (R1 = 1mm)	1.50 (1.01 – 2.21)	0.044
Molecular Subtype (Squamous)	1.54 (1.04 – 2.28)	0.032

Supplementary Table 5: Classical subtype multivariate survival analysis *Final Model*		
	Univariate Analysis	
	HR (95% CI)	*P*
Tumor Grade (High vs low)	2.26 (1.44 – 3.54)	<0.001
Margin Involvement ($R1_{<1mm}$)	2.10 (1.40 – 3.17)	<0.001
Lymphovascular Invasion (Positive)	2.46 (1.62 – 3.73)	<0.001
T stage (8th edition) T2	1.15 (0.61 – 2.16)	0.665
T stage (8th edition) T3	2.23 (1.14 – 4.35)	0.019
Adjuvant Chemotherapy	0.62 (0.40 – 0.95)	0.028

Supplementary Table 6: Squamous subtype multivariate survival analysis *Final Model*		
	Univariate Analysis	
	HR (95% CI)	*P*
Perineural Invasion (Positive)	1.98 (0.89 – 4.40)	0.095
Tumor Location (Body/Tail vs Head)	2.46 (1.17 – 5.17)	0.018
Adjuvant Chemotherapy	0.55 (0.29 – 1.07)	0.079

Supplementary Table 7: The association of adjuvant therapy and molecular subtype (only showing for those with known accurate adjuvant data)			
	Classical Pancreatic *n* = 261 (%)	Squamous *n* = 82 (%)	*P*-value (Chi-square)
Adjuvant Chemotherapy			
Yes	194 (74.3)	51 (62.2)	0.025
No	67 (25.7)	31 (37.8)	
Chemotherapy Agent			
Gemcitabine based	4 (3.5)	2 (6.3)	0.396
Other	109 (96.5)	30 (93.8)	

Supplementary Table 8: The association of neoadjuvant therapy and molecular subtype (only showing for those who received neoadjuvant therapy)			
	Classical Pancreatic *n* = 13 (%)	Squamous *n* = 3 (%)	*P*-value (Chi-square)
Neoadjuvant Therapy			
FOLFIRINOX	0 (0.0)	1 (33.3)	0.376
Gemcitabine	4 (30.8)	0 (0.0)	
Gemcitabine / 5FU ChemoRad	3 (23.1)	1 (33.3)	
Gemcitabine / Abraxane	3 (23.1)	0 (0.0)	
Unknown	3 (23.1)	1 (33.3)	

Supplementary Figures

Figure Legends

Supplementary Figure 1: Kaplan-Meier survival curves for all patient cohorts stratified by (a – d) T-stage, (e – h) N-stage according to the AJCC 8th staging criteria and (i – l) resection margin status. The Verona and Milan cohorts were combined

Supplementary Figure 2: Kaplan-Meier survival curves for all patients stratified by AJCC 8th staging criteria T-status (a - b), lymph node status (c - d) in both margin negative (a, c) and positive (b, d) patients.

Supplementary Figure 3: Kaplan-Meier survival curves for the molecular subtype cohort stratified by molecular subtype (squamous vs classical pancreatic) in both Margin negative (a) and margin positive (b) groups. Margin status defined as $R1_{\leq 1mm}$. Molecular subtype is prognostic in both margin positive and negative groups.

Supplementary Figure 4: Kaplan-Meier survival curves for the molecular subtype cohort stratified by lymph node involvement and margin status $R1_{0mm}$. Lymph node involvement is prognostic in (a) the entire cohort and (b) the Classical Pancreatic subtype, but not (c) in the Squamous subtype. Positive resection margin is prognostic in (d) the entire cohort and (e) the Classical Pancreatic subtype, but not in (f) the Squamous subtype.

Supplementary Figure 5: Kaplan-Meier survival curve for the molecular subtype cohort stratified by administration of adjuvant therapy. Irrespective of adjuvant therapy, squamous subtype had significantly worse prognosis.

Supplementary Figure 6: Kaplan-Meier survival curves for the molecular subtype stratified by S100A2 / A4 expression, lymph node status (a – c) and margin status (d-f). Margin status defined as $R1_{\leq 1mm}$. Biomarker status was determined using immunohistochemistry, and defined as both negative, either S100A2 or A4 positive or both biomarkers positive.

251658240

Supplementary Figure 1

251658240

Supplementary Figure 2

251658240

Supplementary Figure 3

251658240

Supplementary Figure 4

251658240

ChartDescription automatically generated

Supplementary Figure 5

Chart, line chartDescription automatically generated

251658240

Supplementary Figure 6
